# An autopsy case of unexpected death due to Addison’s disease caused by adrenal tuberculosis

**DOI:** 10.1186/s40001-021-00611-w

**Published:** 2021-12-04

**Authors:** Nan Zhao, Yutong Gao, Chunsheng Ni, Danfang Zhang, Xiulan Zhao, Yanlei Li, Baocun Sun

**Affiliations:** 1 Department of Pathology, Tianjin Medical University, No.22 Qixiangtai Road, Heping District, Tianjin, 300070 PR China; 2 Department of Pathology, General Hospital of Tianjin Medical University, Tianjin, 300052 China

**Keywords:** Adrenal crisis, Pulmonary tuberculosis, Adrenal tuberculosis

## Abstract

**Background:**

Adrenal tuberculosis is difficult to diagnose due to non-specific symptom. Unexpected death due to adrenal insufficiency after trauma surgery is rare.

**Case presentation:**

A 45-year-old man, who was admitted to hospital because of trauma to the right hand, died unexpectedly on the 13th day after replantation of amputated fingers. He was diagnosed with brain edema and diluted hyponatremia. Autopsy and histopathologic examination revealed severe brain edema combined with cerebellar tonsillar hernia, extensive destruction of adrenal gland caused by bilateral adrenal tuberculosis and right lung invasive pulmonary tuberculosis.

**Conclusions:**

Trauma and pulmonary tuberculosis complicated with adrenal tuberculosis induced the adrenal crisis, which eventually lead to severe cerebral edema and hernia, and finally death from respiratory and circulatory failure. This autopsy and histopathologic examination suggested a possible pathophysiologic mechanism of sudden death due to diluted hyponatremia after trauma surgery.

## Introduction

Thomas Addison first described the clinical presentation of primary adrenocortical insufficiency in 1855 and Trousseau defined an adrenal insufficiency as an Addison’s disease (AD) in 1856 [1, 2]. Adrenal insufficiency (AD) continues to present a challenge for patients, their physicians and researchers [3]. Tuberculosis was the most common cause of adrenal insufficiency in the developing countries [4]. According to a retrospective analysis of autopsies and adrenalectomies reviewed by K Y Lam and C Y Lo, the adrenal gland was the only extra-pulmonary organ involved by tuberculosis in 14 of these 55 patients (25%; 35 men and 20 women).They found that unexpected and extra-pulmonary tuberculosis such as adrenal tuberculosis has been a common problem [5]. Adrenal tuberculosis is difficult to diagnose due to non-specific symptoms [6]. The diagnosis is therefore often delayed. The fact that adrenal insufficiency may result not only in poor general condition of the patient, but also death due to adrenal crisis makes it all the more important to address this issue seriously [7]. Evidence on clinical presentation and evaluation of patients with adrenal insufficiency caused by bilateral adrenal tuberculosis is scarce.

Here, we report an unusual case of a patient admitted to hospital because of trauma without obvious clinical symptoms and signs of tuberculosis and adrenal insufficiency who died unexpectedly on the 13th day after finger replantation. This autopsy and histopathologic examination suggested a possible pathophysiologic mechanism of sudden death due to adrenal crisis caused by tuberculosis and trauma infection.

## Case presentation

A 45-year-old man, who was urgently admitted to hospital due to bleeding and dysfunction of right hand caused by trauma of the 2nd to 4th fingers. He had an unremarkable medical history and was treated by finger replantation after admission. However, on the 13th day after replantation, the patient fell down after vomiting. The blood pressure (104/70 mmHg, reference range: 90–130/60–85 mmHg) and glucose levels (3.9 mmol/L, reference range: 3.5–5.3 mmol/L) were normal, while head computed tomography (CT)-scan showed cerebral edema and laboratory examination detected an electrolyte disturbance (Table 1). He was diagnosed with brain edema, diluted hyponatremia and bacterial infection. After seizure, respiratory and cardiac arrest suddenly occurred. Although an aggressive resuscitation attempt was made, he was pronounced dead. To investigate the cause of death, a pathologic autopsy was performed 48 h later.

**Table 1:** Laboratory values

Laboratory test	Value	Reference range
pH	7.087	7.350–7.450
Sodium	108.2 mmol/L	136.0–145.0 mmol/L
Calcium	1.030 mmol/L	1.150–1.330 mmol/L
Lactic acid	9.1 mmol/L	0.6–1.4 mmol/L
Chlorine	78.7 mmol/L	98.0–107.0 mmol/L
C-reactive protein	15.03 mg/L	0.00–10.00 mg/L
Procalcitonin	0.73 ng/L	0.04–0.50 ng/L
Cortisol	< 1.00 ug/dL	5.00–25.00 ug/dL
Adrenocorticotropic hormone	855.00 pg/mL	0.00–46.00 pg/mL

At autopsy, the patient was emaciated with dark skin, but without typical hyperpigmentation in the mucosal surfaces. The right hand was swollen with surgical suture and Kirschner wire in the 2nd to 4th fingers; no obvious purulent was present; there were no injuries and surgical incisions in other parts of the body.

**Fig.1:**
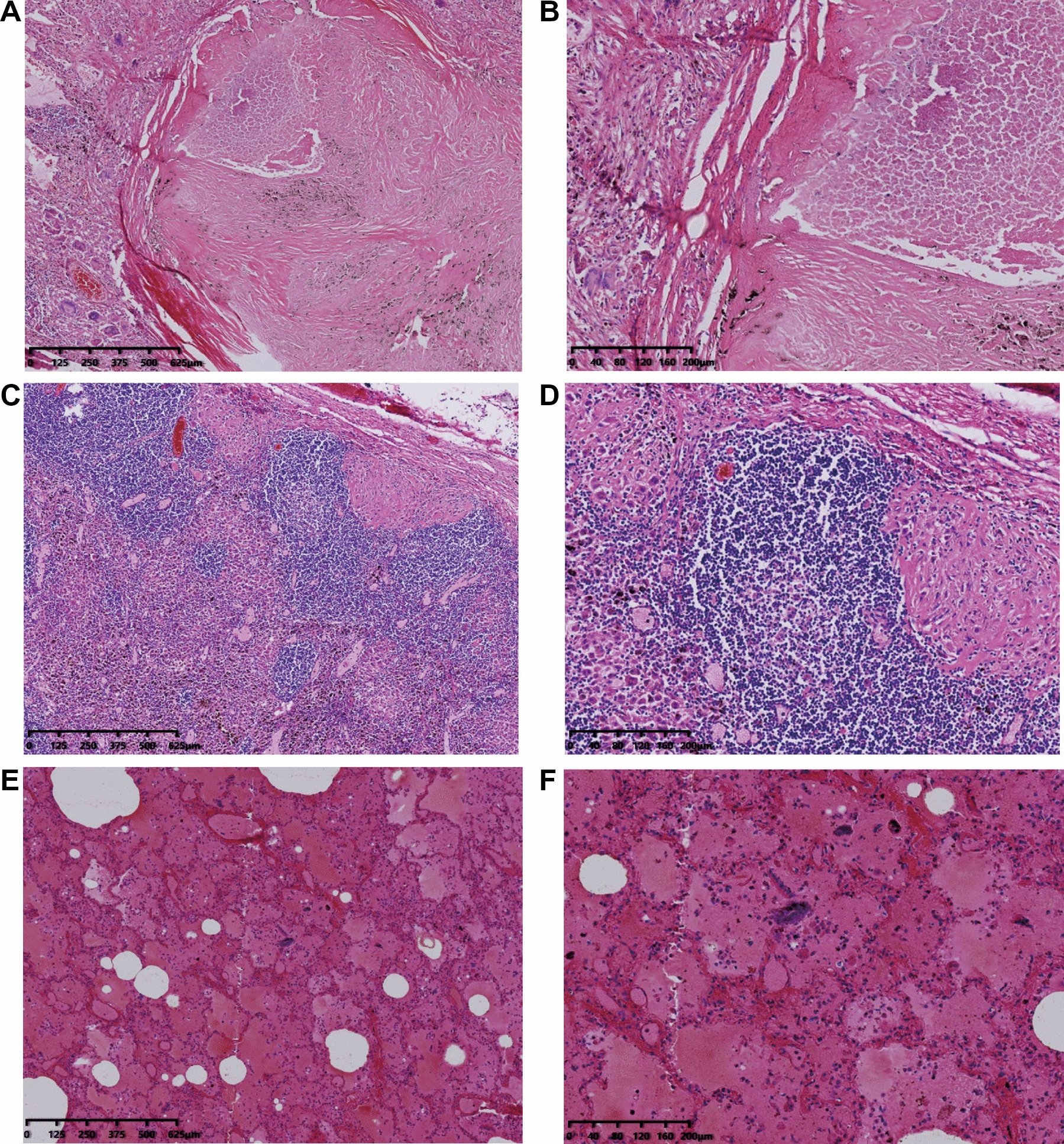
**A** (HE 4 ×) and **B** (HE 10 ×) Tuberculosis of right lung. **C** (HE 4 ×) and **D** (HE 10 ×) Tuberculosis of pulmonary hilar lymph node. **E** (HE 4 ×) and F (HE 10 ×) Congestion and swelling in pulmonary

There was congestion and swelling in the pulmonary bed; calcification foci (the range is about 1.5 × 1 × 1 cm) can be found in the lower lobe of right lung. Pleural thickening of both lungs was observed. Pulmonary hilar lymph nodes were enlarged. Microscopic examination showed congestion in pulmonary bed and swelling in the alveoli of both lungs, especially the lower lobe of right lung. It also showed caseous necrosis with calcification and granuloma formation in the lower lobe of right lung, pleural fibrous tissue hyperplasia of bilateral lung and granulomatous inflammation of hilar lymph nodes (Fig. 1).

**Fig.2:**
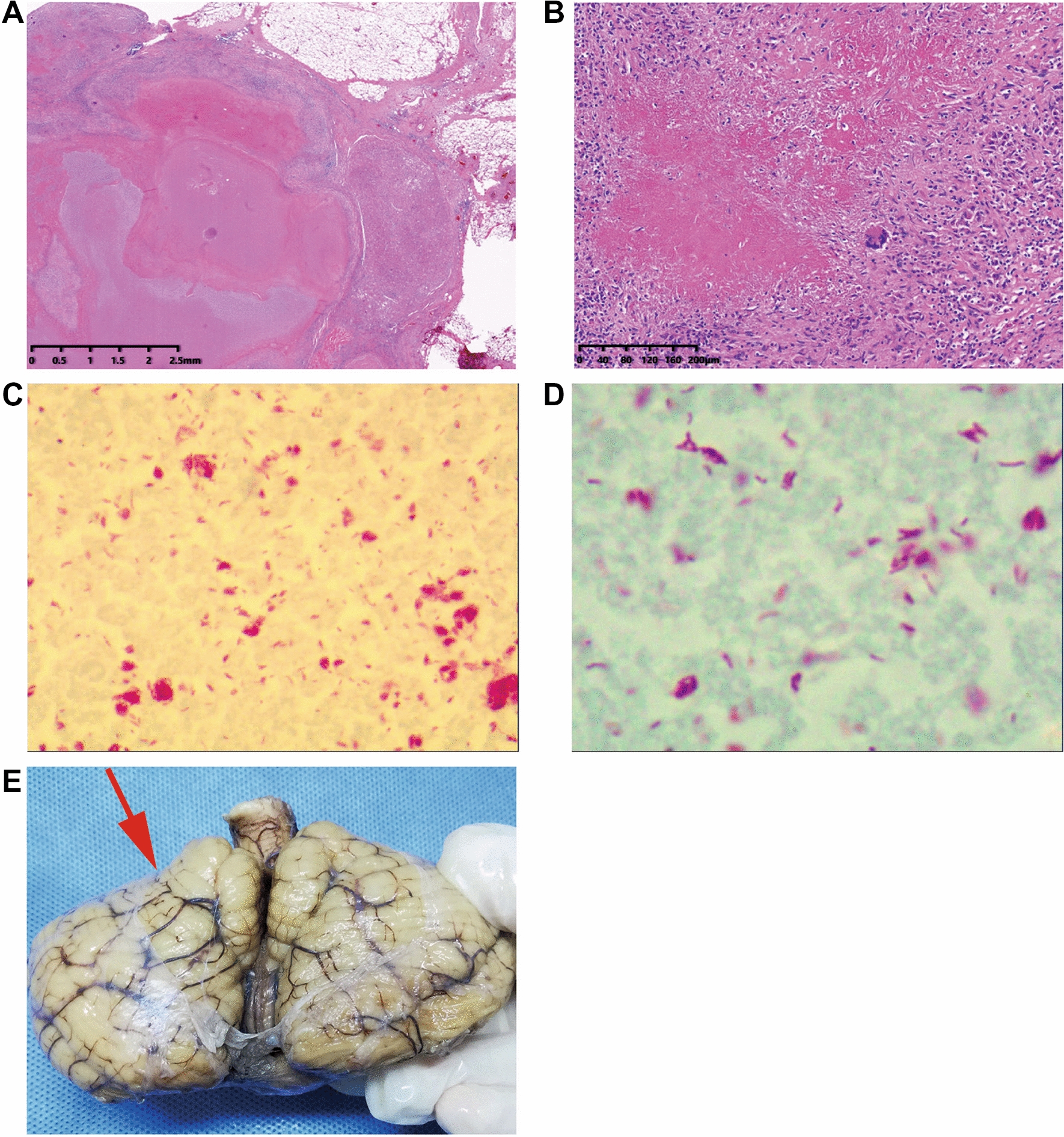
**A** (HE 1 ×) and **B** (HE 10 ×) Irregular nodules with caseous necrosis result in extensive destruction of left adrenal gland. **C** (anti-acid staining 10 ×) and **D** (anti-acid staining 100 ×) Tuberculosis of left adrenal gland. **E** Cerebellar tonsillar hernia (red arrow)

The left adrenal gland was 3 × 2 × 1.5 cm in size and 13.0 g in weight, shown in irregular nodular shape with extensive caseous necrosis and a few residual adrenal gland. There was no obvious right adrenal gland. Microscopic examination showed caseous necrosis with calcification in the left adrenal gland area, anti-acid staining was positive; granulomatous lesions were observed in the right adrenal area, and a little caseous necrosis can be seen in the center (Fig. 2).

The brain was 19 × 15 × 6 cm in size and the cerebellum was 10 × 8 × 4 cm in size. The brain and cerebellum weighed 1446.5 g. Vasodilation and congestion were observed on the surface of meninges; cerebral gyrus was wide and cerebral sulcus was narrow; there was no subarachnoid hemorrhage; both sides of the brain were symmetrical, without bleeding on the section. There were obvious indentation on the right cerebellar tonsils; no bleeding was found in cerebellar section (Fig. 2E). Microscopic examination showed dilation and hyperemia of the blood vessel in subarachnoid, and the perivascular lymphatic space in the brain parenchyma was significantly widened. The nerve fibers were sparse, and the neuron cells were degenerated, showing obvious brain edema changes.

There were no remarkable abnormalities in heart, liver, kidney, spleen and gastrointestinal tract.

## Discussion

We report a patient with tuberculosis-induced adrenal insufficiency (AD), who presented with abnormal electrolytes, but without typical clinical symptoms so that it was ignored by the patient himself. The patient denied medical history when he was brought to hospital because of trauma.

Except for salt craving, the symptoms of primary adrenal insufficiency are rather nonspecific and include weakness, fatigue, musculoskeletal pain, weight loss, abdominal pain, depression and anxiety [6]. As a result, the diagnosis is frequently delayed, resulting in a clinical presentation with an acute life-threatening adrenal crisis [8]. Majority cases of adrenal insufficiency (AD) in the developed countries are attributed to autoimmune adrenalitis in contrast to the developing countries where tuberculosis is implicated to be the most common cause of adrenal insufficiency [7, 9]. In 2019, tuberculosis remained the most common cause of death from a single infectious pathogen [10]. In China, although the incidence of tuberculosis has decreased in recent years, the country still shares a significant part of the global burden of tuberculosis cases (8.4%) [10]. People living in rural areas, those who were less educated showed a low level of awareness of key knowledge about tuberculosis, leading to a delay in seeking care [11]. Adrenal insufficiency which may be caused by adrenal tuberculosis is a severe and potentially life-threatening condition related to the central role of glucocorticoids and/or mineralocorticoids in energy, salt, and fluid homeostasis [12]. Laboratory value of the patient in this case showed significant reduction of cortisol and incredible increase of adrenocorticotropic hormone.

We concluded that trauma and pulmonary tuberculosis complicated with adrenal tuberculosis induced the adrenal crisis, which eventually lead to severe cerebral edema and hernia, and finally death from respiratory and circulatory failure. This autopsy and histopathologic examination suggested a possible pathophysiologic mechanism of sudden death due to diluted hyponatremia after trauma surgery.

## Data Availability

All data generated or analyzed during this study are included in this published article.

## Abbreviations

CT: Computed tomography
H&E: Hematoxylin and eosin
